# Genetic Susceptibility on CagA-Interacting Molecules and Gene-Environment Interaction with Phytoestrogens: A Putative Risk Factor for Gastric Cancer

**DOI:** 10.1371/journal.pone.0031020

**Published:** 2012-02-24

**Authors:** Jae Jeong Yang, Lisa Y. Cho, Kwang-Pil Ko, Aesun Shin, Seung Hyun Ma, Bo Youl Choi, Dong Soo Han, Kyu Sang Song, Yong Sung Kim, Jong-Young Lee, Bok Ghee Han, Soung-Hoon Chang, Hai-Rim Shin, Daehee Kang, Keun-Young Yoo, Sue K. Park

**Affiliations:** 1 Department of Preventive Medicine, Seoul National University College of Medicine, Seoul, Korea; 2 Cancer Research Institute, Seoul National University, Seoul, Korea; 3 Department of Preventive Medicine, Gachon University of Medicine and Science, Incheon, Korea; 4 Cancer Epidemiology Branch, National Cancer Center, Goyang-si, Korea; 5 Department of Preventive Medicine, Hanyang University College of Medicine, Seoul, Korea; 6 Department of Internal Medicine, Hanyang University College of Medicine, Seoul, Korea; 7 Department of Pathology, Chungnam National University College of Medicine, Daejeon, Korea; 8 Medical Genomics Research Center, Korea Research Institute of Bioscience and Biotechnology, Daejeon, Korea; 9 Center for Genome Science, Korea National Institute of Health, Osong, Korea; 10 Department of Preventive Medicine, Konkuk University, Chungju, Korea; 11 Non Communicable Diseases and Health Promotion, World Health Organization, Western Pacific Regional Office, Manila, Philippines; 12 Department of Biomedical Sciences, Seoul National University Graduate School, Seoul, Korea; Florida International University, United States of America

## Abstract

**Objectives:**

To evaluate whether genes that encode CagA-interacting molecules (*SRC*, *PTPN11*, *CRK*, *CRKL*, *CSK*, *c-MET* and *GRB2*) are associated with gastric cancer risk and whether an interaction between these genes and phytoestrogens modify gastric cancer risk.

**Methods:**

In the discovery phase, 137 candidate SNPs in seven genes were analyzed in 76 incident gastric cancer cases and 322 matched controls from the Korean Multi-Center Cancer Cohort. Five significant SNPs in three genes (*SRC*, *c-MET* and *CRK*) were re-evaluated in 386 cases and 348 controls in the extension phase. Odds ratios (ORs) for gastric cancer risk were estimated adjusted for age, smoking, *H. pylori* seropositivity and CagA strain positivity. Summarized ORs in the total study population (462 cases and 670 controls) were presented using pooled- and meta-analysis. Plasma concentrations of phytoestrogens (genistein, daidzein, equol and enterolactone) were measured using the time-resolved fluoroimmunoassay.

**Results:**

*SRC* rs6122566, rs6124914, *c-MET* rs41739, and *CRK* rs7208768 showed significant genetic effects for gastric cancer in both the pooled and meta-analysis without heterogeneity (pooled OR = 3.96 [95% CI 2.05–7.65], 1.24 [95% CI = 1.01–1.53], 1.19 [95% CI = 1.01–1.41], and 1.37 [95% CI = 1.15–1.62], respectively; meta OR = 4.59 [95% CI 2.74–7.70], 1.36 [95% CI = 1.09–1.70], 1.20 [95% CI = 1.00–1.44], and 1.32 [95% CI = 1.10–1.57], respectively). Risk allele of *CRK* rs7208768 had a significantly increased risk for gastric cancer at low phytoestrogen levels (*p interaction*<0.05).

**Conclusions:**

Our findings suggest that *SRC*, *c-MET* and *CRK* play a key role in gastric carcinogenesis by modulating CagA signal transductions and interaction between *CRK* gene and phytoestrogens modify gastric cancer risk.

## Introduction

*Helicobacter pylori* (*H. pylori*), a group I human gastric carcinogen by the International Agency for Research on Cancer (IARC) [1], is the strongest risk factor in the gastric cancer development, and persistent *H. pylori* infection is the first step towards gastric carcinogenesis [1]–[3]. In spite of numerous evidence that *H. pylori* plays a crucial role in gastric carcinogenesis, only a small portion of infected people develop gastric cancer. This implies that other factors involved in the pathogenic mechanism of *H. pylori* can modify individual susceptibility for gastric cancer. Our previous studies demonstrated *H. pylori* infection itself was not associated with the gastric cancer risk but specifically CagA positive *H. pylori* infection significantly increased risk for gastric cancer by 3.57-fold [4], [5].

Cytotoxin-associated gene A (CagA), an immunodominant protein secreted by *H. pylori*, appears to be one of the pathogenic modifying factors [6]–[8]. After infecting *H. pylori* into gastric epithelial cells, CagA acts as a major carcinogenic and virulent component through sequential CagA signal transduction pathway. The first step begins with the interaction between CagA and diverse proteins such as SRC, SHP2, CRK, CRKL, and CSK after phosphorylation and c-MET and GRB2 without phosphorylation [9]–[12]. Tyrosine phosphorylated CagA by SRC family kinases interacts with SHP2 tyrosine phosphatase (encoded by the *PTPN11* oncogene), CRK, CRKL and CSK and induces cell scattering, dissociation and mortality connected to cancer development [8], [12]–[15]. Additionally, non-phosphorylated CagA interacts with c-MET and GRB2 which promotes oncogenic response including cell proliferation and morphological changes such as hummingbird formation [8], [16]–[19]. This CagA translocation and its cellular interaction with those proteins can be a crucial initiating step in gastric carcinogenesis [9]–[12].

Cellular alteration in the CagA positive *H. pylori* pathogenic mechanism appears to explain different susceptibility of gastric cancer among *H. Pylori* infected persons. Since cellular functions can be regulated by their host genes, genetic variants related to the CagA interacting molecules may be the key for individual gastric cancer susceptibility. Based on the putative genetic differences, we hypothesized that genes which encode CagA-interacting proteins may modify risk for gastric cancer. Moreover, we focused on phytoestrogens as an effect modifier in the CagA signal transduction process. Studies have reported that phytoestrogens with anti-inflammatory, anti-bacterial and anti-oxidant properties can inhibit *H. pylori* activity and gastric cancer cell growth and proliferation [20]–[22]. Especially, genistein, one of phytoestrogens and phosphotyrosine kinase inhibitors, is reported to be an effective blocker for CagA phosphorylation [23].

To evaluate the hypotheses, a two-stage genetic analysis that focused on genes which directly encode CagA-binding molecules, *SRC*, *PTPN11*, *CRK*, *CRKL*, *CSK*, *c-MET* and *GRB2*, was conducted that included: 1) the discovery phase that screened and identified single nucleotide polymorphisms (SNPs) with a significant genetic association on gastric cancer; 2) the extension phase that re-analyzed the most significant SNPs in the discovery phase. Additionally, in a sub-analysis, we evaluated the gene-environment interaction to determine whether phytoestrogen levels modify the association between gene polymorphisms which directly encode CagA-binding molecules and risk of gastric cancer.

## Methods

### Ethics Statement

The study protocols were approved by the Institutional Review Board of Seoul National University Hospital (H-0110-084-002 for the KMCC study and C-0910-049-297 for the current nested case-control study) and by the Institutional Review Board of Hanyang University Hospital (2003–4). Moreover, all participants signed an informed consent form before entering the studies.

### Study population

Two-phase genetic association study was conducted. The nested case-control study population was recruited from the Korean Multi-Center Cancer Cohort (KMCC). Detailed information about the KMCC is described elsewhere [24]. Briefly, participants were recruited from four urban and rural areas (Haman, Chungju, Uljin, and Youngil) in Korea. Information on individual characteristics including general lifestyle and environmental exposure was collected using standardized interview-based questionnaires. Blood and spot urine samples were also collected. All participants were passively followed-up through computerized record linkages to the national cancer registry, the national death certificate, and the health insurance medical records. The passive follow-up methods of the KMCC have been reported to be highly efficient and complete [25].

On December 2002, a total of 136 gastric cancer cases defined according to the International Statistical Classification of Diseases and Related Health Problems 10th Revision (ICD-10, C16) were identified in the discovery phase. Among them, 84 cases excluding cases diagnosed before recruitment (n = 36) and without blood samples (n = 16) were initially selected for genotyping. Four cancer-free controls (n = 336) were matched to each gastric cancer case by incidence density sampling based on age (±5 years), sex, residential district, and enrollment year. Eight cases and 14 controls were excluded due to poor genotyping performance, and thus, 76 cases and 322 controls were included in the discovery phase.

In the extension phase, 388 gastric cancer case-control sets were selected as follows: 1) 334 gastric cancer cases including 136 cases identified on December 31, 2002 were ascertained from the KMCC in December 31, 2008. Excluding the cases analyzed in the discovery phase (N = 84) and without blood samples (N = 51), 199 gastric cancer cases were matched 1∶1 to controls according to age (±5 years), sex, and enrollment year. 2) 189 newly diagnosed gastric cancer cases at Chungnam University Hospital and Hanyang University GURI Hospital with informed consent were recruited from March 2002 to September 2006. Blood samples were collected at the time of diagnosis or prior to gastric cancer surgery. Additionally, 189 community-based controls matched by age (±5 years), sex and enrollment year (from 2001 to 2005) were randomly selected from the KMCC. Of the 388 gastric cancer case-control matches, two cases and 40 controls were eliminated due to poor genotyping and insufficient sample and finally, 386 cases and 348 controls were analyzed in the extension phase.

### Candidate genes and SNP selection

In the CagA signal transduction pathway, CagA directly binds to seven proteins that lead to sequential processes. Host genes encoding the seven proteins were selected as follows: v-Crk sarcoma virus CT10 oncogene homolog (*CRK*); v-Crk sarcoma virus CT10 oncogene homolog (avian)-like (*CRKL*); c-Src tyrosine kinase (*CSK*); growth factor receptor-bound protein 2 (*GRB2*); Met proto-oncogene (*c-MET*); nuclear factor of activated T-cells, protein tyrosine phosphatase, non-receptor type 11 (*PTPN11*) encoding SHP2 tyrosine phosphatase and v-Src sarcoma (Schmidt-Ruppin A-2) viral oncogene homolog (*SRC*).

Candidate SNPs were selected according to the three criteria: SNPs reported to have 1) a possible functional relevance for cancer in previous studies; 2) minor allele frequency (MAF) >0.05 in Asian population in public databases such as SNP500Cancer or the international HapMap project using dbSNP IDs (http://www.ncbi.nlm.nih.gov/snp); and concurrently 3) MAF >0.05 in Japanese (JET) in the international HapMap project. Finally, 137 SNPs with a design score = 1.1 and r^2^>0.8 were genotyped to screen the significant SNPs for gastric cancer risk. 108 SNPs are located in the intron region; 24 SNPs are located in the promoter region (flanking region or UTR); five SNPs are located in the coding region.

### Genotyping

In the discovery phase, 137 SNPs in seven candidate genes encoding CagA interacting proteins were genotyped. After measuring concentrations of genomic DNA for all study subjects by a spectrophotometer (NanoDrop ND-1000, NanoDrop Technologies), genotyping was performed using GoldenGate™ assay (Illumina®, San Diego, CA, USA). To ensure quality control and evaluate the intra-subject concordance rate, 52 duplicate samples were randomly distributed in the genotyping plate. Concordance rates for all assays were greater than 99%. Of the 137 SNPs, 21 SNPs were dropped out due to failure of genotyping (4 SNPs), SNP call rate <90% (7 SNPs), HWE <0.0001 (1 SNPs) and MAF ≤0.05 (9 SNPs). Eight cases and 14 controls were also excluded due to genotyping call rate <90%. Finally, 116 SNPs in seven genes (genotyping rate of 99.6%) in 76 cases and 322 controls were analyzed.

In the extension phase, five SNPs with a raw *p-*value <0.02, tag SNPs or higher design scores (rs6122566 and rs6124914 in *SRC*; rs41739 and rs41737 in *c-MET*; rs7208768 in *CRK*) identified in the discovery analysis were genotyped using the Illumina VeraCode GoldenGate Assay with BeadXpress according to the manufacturer's protocol (Illumina®, USA) [26]. To ensure the reliability of the genotyping methods in the two phases, 188 samples were genotyped twice by each method. The concordance rate was >98.4%. Two cases and 40 controls with insufficient DNA (n = 15) or genotyping call rate <90% (n = 27) were excluded. Finally, five SNPs in three genes (genotyping rate of 99.6%) were analyzed in 386 cases and 348 controls.

### *H. pylori* infection and CagA seropositivity

*H. pylori* infection and CagA seropositivity were evaluated using immunoblot assay, Helico Blot 2.1™ (MP Biomedicals Asia Pacific, Singapore). Helico Blot 2.1™ kits have been reported to have high sensitivity and specificity (for sensitivity, 99% identically in both; for specificity, 98% and 90%, respectively) [27].

### Measurements of Phytoestrogen biomarkers

Plasma concentrations of four phytoestrogen biomarkers that were 1) isoflavones: genistein, daidzein, and equol (daidzein metabolite) and 2) lignan: enterolactone were measured using time-resolved fluoroimmunoassay kits (Labmaster, Finland). After free phytoestrogen biomarkers were extracted from 200 µL of plasma sample, the VICTOR3™ 1420 Multilabel Counter measured time-resolved fluorescence (Perkin-Elmer). Detailed measurement methods for phytoestrogen biomarkers are described elsewhere [28]. Of the total study population, plasma concentrations of the four biomarkers were measured in 406 cases and 417 controls with sufficient plasma volume (>200 µL).

### Statistical analysis

To compare the basic characteristics between gastric cancer cases and controls, the chi-square test and Student *t-*test were conducted. *P*-values for difference in proportion for sex, age, *H. pylori* infection, CagA and VacA seropositivity, cigarette smoking, alcohol drinking, and gastritis history between cases and controls were determined.

Hardy-Weinberg equilibrium (HWE) in the control group was evaluated using the chi-square test or Fisher's exact test with a cut-off level of HWE <0.0001. In the discovery phase, minimum global *p*-values (*p*<0.05) in the likelihood ratio test (LRT) with 1 degree of freedom (df) in the additive model and LRT with 2 df in the genotypic model were calculated to select significant SNPs. Using three genetic models, additive, recessive and dominant models, the association between the selected SNPs and gastric cancer risk was analyzed. Permutated *p*-values were estimated by 100,000 permutation tests in the single SNP analysis. To avoid spurious associations with false positive outcomes, the corrected permutated *p*-values on the condition of multiple SNPs and the false discovery rate (FDR) using a Benjamini-Hochberg Method were computed [29]. Gastric cancer risk was estimated as odds ratios (ORs) and 95% confidence intervals (CIs) using unconditional logistic regression model adjusting for risk factors that were age, smoking status (ever *vs.* never), *H. pylori* infection (positive *vs.* negative) and CagA seropositivity (positive *vs.* negative). Additionally, haplotype analysis was performed for genes containing significantly associated SNPs from an individual SNP analysis using Haploview 4.1 software (www.broad.mit.edu/mpg/haploview/).

In the extension phase, the most significant SNPs identified in the discovery phase were re-evaluated. Based on the additive or recessive models, gastric cancer risk was estimated as ORs and 95% CIs using unconditional logistic regression model adjusting for the same covariates mentioned above. To summarize the results from the discovery and the extension phases, pooled- and meta-analysis were conducted. Using the fixed effect model, summarized ORs and 95% CIs were computed. Also, heterogeneity across the studies was evaluated by the Cochran Q statistics [30].

Using analysis of variance and covariance (ANCOVA) with age, smoking status (ever *vs.* never), *H. pylori* infection (positive *vs.* negative) and CagA seropositivity (positive *vs.* negative) as potential risk factors for gastric cancer, the means of the phytoestrogen biomarker levels between cases and controls were compared. Stratified analysis by high and low levels of phytoestrogen biomarkers (genistein, daidzein, equol and enterolactone) where the cut-off levels were determined by the Spline analysis was conducted using unconditional logistic regression models. Interaction effects between the most significant SNPs and phytoestrogen biomarkers were also computed as ORs and 95% CIs adjusted for age, smoking status (ever *vs.* never), *H. pylori* infection (positive *vs.* negative) and CagA seropositivity (positive *vs.* negative).

All statistical analyses were performed using SAS software version 9.2 (SAS Institute, Cary, North Carolina), and PLINK software version 1.07 (http://pngu.mgh.harvard.edu/purcell/plink) [31]. Meta-analyses were conducted using STATA version 10 (Stata, College Station, TX).

## Results

There was no significant difference between cases and controls according to sex, *H. pylori* infection, CagA/VacA seropositivity, smoking/drinking status and gastric ulcer history in the discovery and extension phases (p>0.05). CagA/VacA seropositivity and the proportion of current smokers were significantly higher among gastric cancer cases in the pooled data (*p* = 0.03, *p*<0.01, *p* = 0.02, respectively) (Table S1).

Of the 116 SNPs in the seven candidate genes encoding CagA interacting proteins analyzed in the discovery phase, 22 SNPs in three genes, *SRC*, *c-MET*, and *CRK*, were significantly associated with gastric cancer (*p*-LRT<0.05). *SRC* rs6122566 significantly increased risk for gastric cancer in the recessive models (OR = 4.90, [95% CI 1.19–14.2]). Thirteen SNPs that were rs41739, rs16945, rs41738, rs6566, rs10435378, rs41737, rs2023748, rs41736, rs41735, rs6951311, rs183642, rs2237717 and rs38859 in *c-MET* gene showed a significant gene-dose effect in the linear trend tests (*p*<0.05). *CRK* rs7208768 had a marginally significant gene-dose effect. 100,000 permutation tests in the single SNP analysis showed *SRC* rs6122566, *c-MET* rs41739 and *CRK* rs7208768 with the most significant permutated *p*-value in each gene (*p*_permutation_ = 0.00284, *p*_permutation_ = 0.00989, *p*_permutation_ = 0.01392, respectively). The marginal significance of the corrected permutated *p*-value was observed for *SRC* rs6122566 (*p* = 0.0918) but all FDR *p*-values in all genetic models were not significant (*p*>0.2) (Table S2).

Haplotype blocks were identified by the LD plot (Figure S1). The largest block was constructed with the most significant SNPs including rs41739, rs6566, and rs41738, but the omnibus *p*-value was not significant (*p*>0.05). Four blocks defined by *SRC* and one block defined by *CRK* did not show statistical significance in the omnibus test. The results of the haplotype analysis did not present information beyond individual SNP results (data not shown).

In the extension phase, two SNPs, rs6122566 and rs6124914, in *SRC* gene and rs7208768 in *CRK* remained significantly associated with an increased risk for gastric cancer (OR = 4.01, [95% CI: 1.62–9.96]; OR = 1.30, [95% CI: 1.00–1.70]; OR = 1.33, [95% CI: 1.08–1.64], respectively). Associations between the SNPs in *c-MET* gene (rs41739 and rs41737) and gastric cancer risk were attenuated. In the combined analysis that included the discovery and extension phases, the risk estimate of *SRC* rs6122566 in the recessive model was significantly associated with gastric cancer in both the pooled and meta-analyses (OR = 3.96, [95% CI: 2.05–7.65]; OR = 4.59, [95% CI: 2.74–7.70], respectively). Moreover, *SRC* rs6124914, *c-MET* rs41739 and *CRK* rs7208768 showed significant gene-dose effects for gastric cancer in both analyses. There was no heterogeneity across the analyses (Cochran Q test, *p*>0.05) (Table S3).

Among a total of 823 subjects (406 cases and 417 controls) who were measured the plasma levels of the four phytoestrogen biomarkers, the overall concentrations of genistein, daidzein and enterolactone in cases were significantly lower than those of the controls (genistein 167.6 nmol/L in cases *vs.* 200.2 nmol/L in controls, *p* = 0.0004; daidzein 91.4 nmol/L in cases *vs.* 131.6 nmol/L in controls, *p*<0.0001; enterolactone 51.0 nmol/L in cases *vs.* 77.7 nmol/L in controls, *p*<0.0001). Overall plasma concentrations of equol, a daidzein metabolite, were lower in cases but not statistically significant (50.3 nmol/L for cases *vs.* 62.2 nmol/L for controls; *p* = 0.0977). In stratified analysis according to phytoestrogen biomarkers, a significant gene-environment interaction was observed in *CRK*. Risk allele of *CRK* rs7208768 had a significantly increased risk for gastric cancer at low phytoestrogen levels. Specifically, the A allele of rs7208768 was associated with a greater risk of gastric cancer at low genistein, daidzein, equol and enterolactone and statistically significant (OR = 1.91, [95% CI: 1.44–2.52] at low genistein; OR = 2.09, [95% CI: 1.46–3.01] at low daidzein; OR = 1.87, [95% CI: 1.26–2.78] at low equol; OR = 1.77, [95% CI: 1.10–2.85] at low enterolactone). The *p*-interaction was significant (*p* = 0.0001, *p* = 0.0013, *p* = 0.0147, *p* = 0.0404, respectively) (Table S4).

Though additional stratified analyses were also conducted to detect an interaction between CagA seropositivity and each gene effect for gastric cancer risk, interactions were not significant in any of the three genes, *SRC*, *c-MET* and *CRK* (data not shown).

## Discussion

CagA-secreting *H. pylori* infection appears to play an important role in gastric carcinogenesis via sequential CagA signal transduction pathway. CagA initially binds to seven protein components to activate aberrant cellular responses that underlie the development of gastric cancer. Since function of the protein can be regulated by their host genes, genes that encode CagA interacting molecules may be able to modify risk for gastric cancer. To evaluate this hypothesis, we genotyped 137 SNPs in seven candidate genes and demonstrated that genetic variants of *SRC* (rs6122566 and rs6124914), *c-MET* (rs41739) and *CRK* (rs7208768) were significantly associated with gastric cancer risk. Additionally, an interactive effect of *CRK* genetic polymorphism, rs7208768, and four phytoestrogen biomarkers, genistein, daidzein, equol and enterolactone on gastric cancer risk were analyzed.

SRC, a non-receptor protein tyrosine kinase (TK), appear to be essential in gastric carcinogenesis. Once injected into gastric epithelial cells, CagA undergoes tyrosine phosphorylation by the SRC family kinases [8], [12], [18]. The tyrosine phosphorylation of CagA is an integral step in determining the sequential cellular signaling mechanism. Because some CagA interacting molecules such as SHP-2, CRK and CSK are only able to respond with phosphorylated CagA, SRC can be more important in influencing other's cellular functions and inducing development of gastric cancer. Additionally, SRC has been reported to play a crucial role in tumor progression and mediate cancer development and metastasis [32]. Cellular activity of SRC appears to be altered by the host gene and our results indicate that *SRC* rs6122566 and rs6124914 can be risk modifiers in gastric carcinogenesis. *SRC* genetic variations that influence the cellular capacity in gastric epithelial cells are associated with gastric cancer risk.

Despite the attenuated significance in the extension analysis, *c-MET* which is synonymous with *HGFR* (hepatocyte growth factor receptor) may be an independent risk gene for gastric cancer. Numerous previous studies reported that c-MET, one of the receptor TKs, promotes invasive tumor growth, cell invasion, and mortality, and amplification and/or overexpression of c-MET was associated with various human carcinoma including gastric cancer [17], [33]–[36]. In terms of c-MET cellular mechanism, CagA plays a role as an adaptor protein, Gab, to mediate receptor TK signaling by controlling a cluster of downstream components at the activated receptor such as Grb2, PLCγ, and SHP-2 [37], [38]. By functionally mimicking the Gab adaptor protein, CagA might stimulate abnormal proliferation and mortality of gastric epitherial cells [39]. In the present study, a polymorphism of *c-MET* gene (rs41739) was significantly associated with gastric cancer risk and a possible genetic susceptible factor on gastric cancer. Consistent with the cellular importance and function, *c-MET* gene appears to modify the risk for gastric carcinogenesis through CagA signal transduction pathway.

CRK adaptor protein which has splicing isoforms, CRK-I (SH2-SH3) and CRK-II (SH2-SH3-SH3), binds to TKs and controls transcription and cytoskeletal reorganization modulating cellular activities [40]. Also, this adaptor protein integrates various cellular signals and its dysregulation is connected to the human carcinoma [41]. Interaction between CRK and phosphorylated CagA has been reported to be a biological prerequisite that leads to morphological change, cell scattering and deregulation of cell–cell adhesion in the gastric epithelium [14]. Several studies have indicated that overexpression of CRK is associated with various types of human cancers including lung, gastric and colon cancer [42], [43]. Our findings also support the genetic potential of *CRK* rs7208768 on the development of gastric cancer and both genetic and cellular magnitude of CRK.

More interestingly, significant interactions between the *CRK* genetic polymorphism and four phytoestrogen biomarkers, genistein, daidzein, equol and enterolactone, modified gastric cancer risk. Studies indicated protective effects of phytoestrogens on gastric cancer [20], [28] and particularly, genistein inhibited the ERK signal transduction cascade induced by *H. pylori* infection playing a role as a tyrosine kinase inhibitor [44]. Considering CRK is the major upstream molecule of ERK activation, the risky genetic variants of *CRK* to activate the ERK signaling can be blocked by phytoestrogens, and mediate the development of gastric cancer.

SRC, c-MET and CRK are also involved in the protein TKs that is a diverse multigene family which controls cellular signal transduction pathway mediating a range of downstream cellular processes and plays significant roles in the development of various clinical diseases [45], [46]. TKs are also known as oncogenes involved in human malignancies. SRC belongs to a non-receptor TK and c-MET is a receptor TK, while CRK is an adaptor protein which binds to TK-phosphorylated proteins and strengthens the main proteins in the signal transduction pathway [41]. These three molecules encoded by *SRC*, *c-MET*, and *CRK* genes can independently induce cell differentiation, adhesion, death and morphological changes by transmiting cell signals related to their TK activities regardless of interaction with CagA. Futhermore, genes related to TK action appear to play a crucial role as a susceptible factor for gastric cancer considering genistein that is a tyrosine kinase inhibitor can reduce gastric cancer risk [28]. This indicates that genetic susceptibilities of *SRC*, *c-MET*, and *CRK* in gastric carcinogenesis should be treated as independent risk factors that modify the cellular signal transduction in TK dependent manners because uninfected persons with CagA secreting *H. Pylori* can be at risk for gastric cancer depending on individual genetic variants of the three genes.

Though *PTPN11*, *CRKL*, *CSK*, and *GRB2* did not show any significant association with gastric cancer in the present study, their genetic effects should not be overlooked. At the cellular level, these molecules are significantly related to aberrant effects that underlie gastric carcinogenesis [12]. As one of the human proto-oncogenes, *PTPN11* encodes cytoplasmic tyrosine phosphatase with SHP2 and can induce aberrant hyperactivation of the ERK signaling [47]. A study has also reported that a *PTPN11* genetic variant increased the risk for gastric atrophy and cancer among CagA positive *H. pylori* infected people [48]. In the CagA signal transduction pathway, CRKL works quite similarly to CRK; CSK frustrates an activity of SRC family kinase and CagA-SHP2 signaling; and GRB2 acts as a trigger to activate the RAS/MEK/ERK pathway [14], [18], [49]. Further studies with a greater number of gastric cancer cases and wider coverage of genetic polymorphisms in these genes are warranted.

Gastric carcinogenesis induced by CagA positive *H. Pylori* infection can be infered from our study results and review of cellular mechanisms [16], [18], [47] (Figure S2). Once CagA is injected in gastric ephithelial cells, SRC initiates CagA phosphorylation that interacts with CRK adaptor protein and SHP2 to promote the ERK activation. Non-phosphorylated CagA mimics the Gab adaptor protein to potentiate the c-MET-HGF intracellular signaling and stimulate c-MET signals that also activates the ERK signal cascade. As a result, CagA binding molecules such as SRC, c-MET, CRK, SHP2, CRKL and GRB2 interacts with phosphorylated or non-phosphorylated CagA to stimulate downstream signals in the ERK activation inducing oncogenic effects on gastric cancer, whereas, CSK inhibits SRC family kinase functions and CagA-SHP2 signaling effects. In terms of genetic mechanism, significant genetic markers of *SRC* (rs6122566 and rs6124914), *c-MET* (rs41739) and *CRK* (rs7208768) are actively involved in gastric carcinogenesis. Interestingly, primary interacting molecules, SRC, c-MET, CRK and SHP2, contribute to mutual development and sequentially connect to operate carcinogenic effect. In particular, SRC can extensively influence the activity of the others from migration to signal transduction [11], [50].

We examined SNPs from our other study that used the Affymetrix 5.0 platform and the Korea Centers for Disease Control and Prevention (KCDC) study that used the Affymetrix 6.0 platform to find consistencies. In the KCDC study, *SRC* rs6122566 which was the most significant SNP in the present study showed a raw *p-*value of 0.0009 in the single SNP analysis. Unfortunately, many other significant SNPs were not included in the platforms due to different SNP selection methods (random SNPs *vs.* candidate SNPs according to MAF >0.05 in Asians) and target populations (Caucasian *vs.* Koreans). Nevertheless, our results might be applicable to most East-Asian populations because the minor allele frequencies of our significant SNPs showed similarities to other Asian populations such as Chinese and Japanese (http://www.hapmap.org) (Appendix S1).

Limitations should be noted. First, due to the restricted number of study subjects, we did not have sufficient statistical power and were not able to perform subgroup analyses for gastric cancer types such as histological (intestinal *vs.* diffuse) and anatomical subtypes (cardia *vs.* non-cardia). Second, we only focused on CagA interacting molecules and thus, secondary interacting molecules in CagA downstream signaling pathways such as RAS and ERK cascade were not included in this study. Third, in the extension phase, selection bias may be induced because hospital-based cases were matched to community-based controls. However, considering that 1) genetic traits are inborn and not easily changeable, 2) all cases were matched to controls according to the major covariates in the initial study design stage, 3) effects of confounding factors were considered by the use of multivariable models, and 4) no heterogeneity between hospital- and community-based cases in meta-analysis, the potential selection bias may be minimized.

In spite of the limitations, this is a two-phase genetic association study that provides evidence on the role of CagA cellular mechanism-related genes. Through the candidate approach of the discovery phase, the most significant SNPs were preliminarily screened and after, the SNPs were re-evaluated in the extension phase. Moreover, through intensive analyses that focused on gene and gene-environment interaction, conclusive evidence is provided to elucidate the etiology of gastric cancer.

This study shows *SRC*, *c-MET*, and *CRK* genetic variants can be susceptible genetic factors for the development of gastric cancer by controlling signals through the CagA transduction pathways. Moreover, an interaction between *CRK* genetic polymorphism and phytoestrogen biomarkers appear to play a role as risk modifiers in gastric carcinogenesis. Replication studies with a greater number of cases and more substantial genomic coverage of the genes will allow us to elucidate gastric cancer pathological mechanisms based on the CagA signal transduction.

## Supporting Information

Appendix S1 **Detailed information on the selected SNPs in CagA transduction pathway associated with gastric cancer (among controls): In the discovery phase.**
(DOC)

Figure S1 **Gene maps and LD blocks.** a. D' and LOD values were used for selection of LD color scheme.
(TIF)

Figure S2 **Schematic diagram of oncogenic effects in CagA signal transduction pathway.** a. SRC initiates CagA phosphorylation. b. Phosphorylated CagA interacts with CRK adaptor protein and SHP2 (encoded by the *PTPN11* gene). c. Non-phosphorylated CagA potentiates c-MET signals and the c-MET-HGF intracellular signaling. d. SRC, c-MET, CRK and SHP2 interacts with phosphorylated or non-phosphorylated CagA to stimulate the ERK cascade linked to aberrant cellular functions that leads to the development of gastric cancer. e. Genetic polymorphisms of *SRC* (rs6122566 and rs6124914), *c-MET* (rs41739) and *CRK* (rs7208768) are significantly associated with gastric cancer risk. f. CSK inhibits SRC family kinase activities and CagA-SHP2 signaling effects. g. Phytoestrogens (Genistein, Daidzein, Equol and Enterolactone) modify the *CRK* genetic effects.
(TIF)

Table S1 **Basic characteristics of gastric cancer cases and controls in the genetic analysis: discovery, extension and pooled analyses.**
(DOC)

Table S2 **Significant SNPs for genes which directly encode CagA-binding molecules associated with gastric cancer in the discovery phase.**
(DOC)

Table S3 **Association between representative SNPs in CagA-binding molecules and gastric cancer risk.**
(DOC)

Table S4 **The gastric cancer risk associated with the interaction between SNPs in CagA-binding molecules and phytoestrogens.**
(DOC)
