# Supplementary material for: Genetic Susceptibility on CagA-Interacting Molecules and Gene-Environment Interaction with Phytoestrogens: A Putative Risk Factor for Gastric Cancer
Source: PLoS One. 2012 Feb 24;7(2):e31020. doi: 10.1371/journal.pone.0031020 (PMC3286459; doi:10.1371/journal.pone.0031020)
Supplement: Appendix S1 — Detailed information on the selected SNPs in CagA transduction pathway associated with gastric cancer (among controls): In the discovery phase. (DOC) [file pone.0031020.s001.doc]

**Appendix**

**Detailed information on the selected SNPs in CagA transduction pathway associated with gastric cancer (among controls): In the discovery phase**

| **CHR** | **GENE** | **Gene description** | **db SNP ID** | **Chromosome**  **position** | **Nucleotide change**  **(protein change)** | **MAF from HapMap database** | | | |  | **The present study in Korean** | | | |
| --- | --- | --- | --- | --- | --- | --- | --- | --- | --- | --- | --- | --- | --- | --- |
|  |  |  |  |  |  | **Allele** | **CHB** | **CHD** | **JPT** |  | **Allele** | **MAF** | **HWE** | **Genoty**  **ping** |
| 17 | CRK | v-Crk sarcoma virus  CT10 oncogene homolog | rs1083 | 1272047 | Flanking_3UTR-143T>C | G | 0.089 | 0.101 | 0.186 |  | C | 0.124 | 0.592 | 1.000 |
|  |  |  | rs11655449 | 1274465 | Intron-771A>C | A | 0.512 | 0.512 | 0.490 |  | A | 0.421 | 0.460 | 0.995 |
|  |  |  | rs11657524 | 1304873 | Intron-1048C>G | G | 0.422 | - | 0.344 |  | G | 0.455 | 0.529 | 0.982 |
|  |  |  | rs16946807 | 1303329 | Intron-2592A>C | A | 0.393 | 0.388 | 0.407 |  | A | 0.456 | 0.598 | 0.984 |
|  |  |  | rs2063187 | 1295739 | Intron-8540A>C | C | 0.089 | 0.100 | 0.186 |  | C | 0.126 | 0.646 | 0.993 |
|  |  |  | rs7208768 | 1299451 | Intron-6470A>G | A | 0393 | 0.376 | 0.407 |  | A | 0.455 | 0.567 | 0.995 |
|  |  |  | rs8073032 | 1283157 | Intron-3507T>C | C | 0.089 | 0.100 | 0.186 |  | C | 0.124 | 0.592 | 1.000 |
| 22 | CRKL | v-Crk sarcoma virus  CT10 oncogene homolog  (avian)-like | rs1043235 | 19636400 | 3UTR[2266/1490]A>G | A | 0.247 | 0.212 | 0.212 |  | A | 0.221 | 0.483 | 1.000 |
|  |  |  | rs1043242 | 19636743 | 3UTR[2609/1147]A>G | A | 0.247 | 0.208 | 0.214 |  | A | 0.221 | 0.483 | 1.000 |
|  |  |  | rs17819409 | 19632320 | Intron-1679T>C | C | 0.417 | 0.394 | 0.401 |  | C | 0.363 | 0.720 | 0.989 |
|  |  |  | rs2266953 | 19632925 | Intron-1074T>C | C | 0.244 | 0.212 | 0.215 |  | C | 0.219 | 0.592 | 0.977 |
|  |  |  | rs2285547 | 19637058 | 3UTR[2924/832]T>C | C | 0.143 | 0.141 | 0.131 |  | C | 0.119 | 0.455 | 1.000 |
|  |  |  | rs3827296 | 19610463 | Intron-7604C>G | G | 0.156 | - | 0.102 |  | G | 0.119 | 0.455 | 1.000 |
|  |  |  | rs5761368 | 19607471 | Intron-4938A>G | A | 0.143 | 0.141 | 0.145 |  | A | 0.121 | 0.499 | 1.000 |
|  |  |  | rs5761386 | 19615846 | Intron-2221T>G | T | 0.143 | 0.141 | 0.145 |  | T | 0.119 | 0.455 | 1.000 |
|  |  |  | rs5761424 | 19623201 | Intron-4669A>G | A | 0.071 | 0.065 | 0.105 |  | A | 0.079 | 0.128 | 1.000 |
|  |  |  | rs737895 | 19638235 | Flanking_3UTR-345C>G | G | 0.156 | - | 0.102 |  | G | 0.121 | 0.230 | 1.000 |
| 15 | CSK | c-Src tyrosine kinase | rs12439525 | 72874458 | Intron-3169C>T | T | 0.378 | - | 0.295 |  | T | 0.333 | 0.062 | 0.968 |
|  |  |  | rs12442901 | 72870965 | Intron-6662A>G | A | 0.427 | 0.417 | 0.476 |  | A | 0.408 | 0.322 | 0.995 |
|  |  |  | rs12595627 | 72870966 | Intron-2378A>G | T | 0.244 | 0.347 | 0.271 |  | T | 0.309 | 0.649 | 0.991 |
|  |  |  | rs1378938 | 72883496 | Flanking_3UTR-938 | T | 0.429 | 0.429 | 0.465 |  | A | 0.421 | 0.172 | 0.995 |
|  |  |  | rs1378940 | 72870547 | Intron-7080T>G | A | 0.156 | - | 0.182 |  | T | 0.167 | 0.672 | 0.984 |
|  |  |  | rs1378941 | 72867203 | Intron-5089T>G | A | 0.143 | - | 0.179 |  | T | 0.173 | 0.355 | 0.984 |
|  |  |  | rs1378942 | 72864420 | Intron-2306A>G | A | 0.137 | 0.119 | 0.209 |  | T | 0.174 | 0.206 | 0.998 |
|  |  |  | rs16972620 | 72866893 | Intron-4779T>C | T | 0.179 | 0.253 | 0.134 |  | T | 0.163 | 0.160 | 0.998 |
|  |  |  | rs16972628 | 72867509 | Intron-5395A>G | G | 0.173 | 0.253 | 0.134 |  | G | 0.163 | 0.157 | 1.000 |
|  |  |  | rs16972714 | 72875076 | Intron-2551C>G | C | 0.186 | - | 0.148 |  | C | 0.173 | 0.348 | 0.995 |
|  |  |  | rs2168519 | 72867925 | Intron-5811A>G | T | 0.417 | 0.412 | 0.471 |  | A | 0.415 | 0.407 | 0.846 |
|  |  |  | rs2229730 | 72880442 | Coding[36/54]T>C | T | 0.173 | 0.112 | 0.145 |  | - | 0.000 | - | 0.000 |
|  |  |  | rs2301249 | 72879437 | Intron-369T>C | T | 0.429 | 0.424 | 0.471 |  | T | 0.422 | 0.185 | 0.848 |
|  |  |  | rs3784789 | 72869605 | Intron-7491C>G | G | 0.167 | - | 0.193 |  | C | 0.184 | 0.001 | 0.986 |
|  |  |  | rs4886409 | 72874932 | Intron-2695T>C | A | 0.339 | 0.359 | 0.279 |  | T | 0.335 | 0.090 | 0.995 |
|  |  |  | rs7085 | 72882536 | 3UTR[628/22]A>G | T | 0.429 | 0.429 | 0.476 |  | T | 0.426 | 0.254 | 0.834 |
|  |  |  | rs8033381 | 72867738 | Intron-5624A>G | G | 0.417 | 0.418 | 0.471 |  | G | 0.422 | 0.434 | 0.855 |
|  |  |  | rs8039046 | 72883405 | Flanking_3UTR-847A>G | G | 0.173 | 0.129 | 0.151 |  | G | 0.212 | 0.410 | 1.000 |
| 17 | GRB2 | growth factor receptor-bound protein 2 | rs2053158 | 70905054 | Intron-3613A>T | T | 0.055 | - | 0.047 |  | T | 0.041 | 0.034 | 0.993 |
|  |  |  | rs2117561 | 70843046 | Flanking_5UTR-2573T>G | C | 0.078 | - | 0.045 |  | G | 0.044 | 0.067 | 0.979 |
|  |  |  | rs2385264 | 70876587 | Flanking_3UTR-24640A>G | C | 0.048 | 0.076 | 0.047 |  | G | 0.046 | 0.000 | 1.000 |
|  |  |  | rs4542691 | 70838560 | Intron-1816T>C | T | 0.089 | 0.129 | 0.058 |  | T | 0.068 | 0.660 | 1.000 |
|  |  |  | rs4788891 | 70902740 | Intron-1299A>G | A | 0.030 | 0.029 | 0.000 |  | A | 0.017 | 0.765 | 0.855 |
|  |  |  | rs4789178 | 70879837 | Flanking_3UTR-21390C>G | C | - | - | - |  | C | 0.043 | 0.062 | 1.000 |
|  |  |  | rs4789181 | 70886235 | Flanking_3UTR-14992A>T | T | 0.067 | - | 0.045 |  | T | 0.043 | 0.062 | 1.000 |
|  |  |  | rs4789182 | 70886540 | Flanking_3UTR-14687A>G | G | 0.089 | 0.129 | 0.058 |  | G | 0.068 | 0.660 | 1.000 |
|  |  |  | rs4789183 | 70891816 | Flanking_3UTR-9411A>G | G | - | - | - |  | G | 0.042 | 0.047 | 0.998 |
|  |  |  | rs7207618 | 70833282 | Intron-292T>C | T | 0.111 | - | 0.067 |  | T | 0.067 | 0.614 | 0.993 |
|  |  |  | rs7219 | 70826963 | 3UTR[1211>1080]A>G | C | 0.101 | 0.155 | 0.052 |  | G | 0.070 | 0.710 | 1.000 |
|  |  |  | rs8079197 | 70828274 | Intron-45C>G | G | 0.111 | - | 0.068 |  | G | 0.068 | 0.660 | 1.000 |
|  |  |  | rs959260 | 70881017 | Flanking_3UTR-20210A>G | C | 0.048 | 0.082 | 0.047 |  | G | 0.043 | 0.062 | 1.000 |
|  |  |  | rs9900002 | 70893613 | Flanking_3UTR-7614A>T | T | - | - | - |  | T | 0.044 | 0.062 | 0.998 |
| 7 | c-MET | Met proto-oncogene | rs10234854 | 116131947 | Intron-4373A>G | A | 0.318 | - | 0.279 |  | A | 0.312 | 0.207 | 0.991 |
|  |  |  | rs10243024 | 116133839 | Intron-6265A>G | A | 0.244 | 0.282 | 0.262 |  | A | 0.255 | 0.479 | 0.968 |
|  |  |  | rs10246585 | 116131334 | Intron-3760T>C | C | 0.311 | - | 0.278 |  | C | 0.312 | 0.229 | 0.991 |
|  |  |  | rs10248537 | 116129863 | Intron-2289T>G | T | 0.311 | - | 0.267 |  | T | 0.311 | 0.220 | 1.000 |
|  |  |  | rs10271561 | 116102441 | Intron-2574T>C | C | 0.214 | - | 0.221 |  | C | 0.269 | 0.468 | 1.000 |
|  |  |  | rs10435378 | 116212729 | Intron-1970A>G | A | 0.488 | 0.512 | 0.448 |  | G | 0.481 | 0.975 | 1.000 |
|  |  |  | rs11767567 | 116102104 | Intron-2237A>G | A | 0.314 | - | 0.263 |  | A | 0.234 | 0.298 | 1.000 |
|  |  |  | rs12539654 | 116117219 | Intron-9142T>C | T | 0.111 | - | 0.033 |  | T | 0.085 | 0.338 | 1.000 |
|  |  |  | rs13223756 | 116184808 | Coding[81/21]A>G | G | 0.232 | 0.276 | 0.238 |  | G | 0.248 | 0.588 | 1.000 |
|  |  |  | rs1621 | 116224842 | 3UTR[1427/834]A>G | G | 0.113 | 0.106 | 0.145 |  | G | 0.140 | 0.742 | 0.995 |
|  |  |  | rs16945 | 116225890 | Flanking_3UTR-214T>C | T | 0.482 | 0.512 | 0.442 |  | C | 0.475 | 0.815 | 0.998 |
|  |  |  | rs17138937 | 116127818 | Intron-244A>C | C | 0.302 | - | 0.278 |  | C | 0.313 | 0.253 | 0.995 |
|  |  |  | rs17138943 | 116129516 | Intron-1942A>G | A | 0.078 | 0.076 | 0.041 |  | A | 0.085 | 0.338 | 1.000 |
|  |  |  | rs17138948 | 116131546 | Intron-3972T>C | T | 0.084 | 0.077 | 0.041 |  | T | 0.085 | 0.338 | 1.000 |
|  |  |  | rs17138978 | 116185999 | Intron-89T>C | T | 0.127 | 0.124 | 0.058 |  | T | 0.135 | 0.676 | 0.998 |
|  |  |  | rs17138983 | 116189969 | Intron-371A>G | G | 0.125 | 0.124 | 0.058 |  | G | 0.132 | 0.795 | 0.991 |
|  |  |  | rs183642 | 116220866 | Intron-2079A>G | G | 0.482 | 0.500 | 0.442 |  | A | 0.474 | 0.733 | 1.000 |
|  |  |  | rs193686 | 116218663 | Intron-4282T>C | C | 0.113 | 0.082 | 0.134 |  | C | 0.114 | 0.286 | 0.987 |
|  |  |  | rs2023748 | 116223258 | Coding[81/156]A>G | G | 0.482 | 0.506 | 0.441 |  | A | 0.475 | 0.815 | 1.000 |
|  |  |  | rs2073560 | 116210397 | Intron-197A>G | A | 0.198 | - | 0.256 |  | A | 0.246 | 0.441 | 1.000 |
|  |  |  | rs2237708 | 116124116 | Intron-2245T>C | C | 0.315 | 0.353 | 0.273 |  | C | 0.316 | 0.217 | 1.000 |
|  |  |  | rs2237709 | 116124380 | Intron-1981A>G | A | 0.311 | - | 0.278 |  | A | 0.303 | 0.490 | 0.986 |
|  |  |  | rs2237710 | 116124588 | Intron-1773T>G | G | 0.363 | 0.435 | 0.302 |  | G | 0.385 | 0.290 | 0.991 |
|  |  |  | rs2237711 | 116125233 | Intron-1128A>C | A | 0.089 | 0.083 | 0.144 |  | A | 0.135 | 0.014 | 1.000 |
|  |  |  | rs2237713 | 116130376 | Intron-2802A>G | A | - | - | - |  | A | 0.073 | 0.287 | 1.000 |
|  |  |  | rs2237717 | 116192623 | Intron-2065T>C | C | 0.488 | 0.494 | 0.442 |  | T | 0.477 | 0.898 | 0.995 |
|  |  |  | rs2283053 | 116214255 | Intron-3496A>G | G | 0.238 | 0.280 | 0.238 |  | G | 0.247 | 0.452 | 0.989 |
|  |  |  | rs2299433 | 116118114 | Intron-8247T>C | T | 0.315 | 0.353 | 0.273 |  | T | 0.314 | 0.289 | 0.998 |
|  |  |  | rs2299435 | 116128557 | Intron-983T>C | C | 0.122 | - | 0.035 |  | C | 0.085 | 0.338 | 1.000 |
|  |  |  | rs2299436 | 116128619 | Intron-1045A>G | G | 0.315 | 0.353 | 0.273 |  | G | 0.313 | 0.253 | 0.995 |
|  |  |  | rs2299437 | 116128724 | Intron-1150A>G | A | 0.315 | 0.357 | 0.276 |  | A | 0.313 | 0.25 | 1.000 |
|  |  |  | rs2299438 | 116128990 | Intron-1416T>G | G | 0.311 | - | 0.278 |  | G | 0.312 | 0.229 | 0.989 |
|  |  |  | rs2299439 | 116203246 | Intron-845T>G | T | 0.482 | 0.500 | 0.440 |  | - | 0.000 | - | 0.000 |
|  |  |  | rs2299440 | 116203563 | Intron-1116T>C | T | 0.232 | 0.276 | 0.238 |  | T | 0.245 | 0.507 | 0.995 |
|  |  |  | rs2402118 | 116215809 | Intron-5050A>C | A | 0.083 | 0.059 | 0.105 |  | A | 0.076 | 0.001 | 1.000 |
|  |  |  | rs2896191 | 116121280 | Intron-5081T>G | T | 0.111 | - | 0.033 |  | T | 0.085 | 0.338 | 1.000 |
|  |  |  | rs3807996 | 116164043 | Intron-3197T>C | T | 0.156 | - | 0.068 |  | T | 0.132 | 0.438 | 1.000 |
|  |  |  | rs3807997 | 116207794 | Intron-1484A>C | A | 0.232 | 0.276 | 0.238 |  | A | 0.246 | 0.441 | 1.000 |
|  |  |  | rs38841 | 116107162 | Intron-7295A>G | G | 0.274 | 0.324 | 0.238 |  | G | 0.262 | 0.812 | 0.995 |
|  |  |  | rs38842 | 116107641 | Intron-7774A>T | A | - | - | - |  | A | 0.334 | 0.141 | 1.000 |
|  |  |  | rs38845 | 116109038 | Intron-9171A>G | A | 0.389 | 0.524 | 0.411 |  | A | 0.399 | 0.903 | 0.982 |
|  |  |  | rs38849 | 116119775 | Intron-6586C>G | C | 0.089 | - | 0.170 |  | C | 0.162 | 0.060 | 0.991 |
|  |  |  | rs38851 | 116130574 | Intron-3000A>C | A | 0.452 | - | 0.477 |  | C | 0.455 | 0.883 | 0.998 |
|  |  |  | rs38852 | 116141761 | Intron-14187A>T | T | 0.363 | 0.372 | 0.413 |  | T | 0.404 | 0.939 | 0.942 |
|  |  |  | rs38854 | 116144447 | Intron-14511T>C | C | 0.444 | - | 0.500 |  | T | 0.471 | 0.572 | 1.000 |
|  |  |  | rs38855 | 116145280 | Intron-13678A>G | A | 0.375 | 0.387 | 0.424 |  | A | 0.396 | 0.563 | 0.995 |
|  |  |  | rs38857 | 116152649 | Intron-6309T>C | T | 0.078 | - | 0.136 |  | T | 0.122 | 0.100 | 1.000 |
|  |  |  | rs38858 | 116157523 | Intron-1435T>G | G | 0.278 | - | 0.409 |  | G | 0.396 | 0.652 | 0.979 |
|  |  |  | rs38859 | 116166330 | Intron-910T>C | C | 0.476 | 0.500 | 0.435 |  | T | 0.484 | 0.942 | 0.995 |
|  |  |  | rs39747 | 116108275 | Intron-8408T> | C | 0.518 | 0.406 | 0.442 |  | C | 0.461 | 0.399 | 0.989 |
|  |  |  | rs39748 | 116114666 | Intron-11695C>G | C | 0.456 | - | 0.489 |  | G | 0.450 | 0.771 | 0.995 |
|  |  |  | rs40238 | 116101563 | Intron-1696A>C | A | - | - | - |  | A | 0.112 | 0.005 | 1.000 |
|  |  |  | rs40239 | 116105113 | Intron-5246A>G | G | 0.196 | 0.176 | 0.209 |  | G | 0.169 | 0.131 | 0.995 |
|  |  |  | rs41735 | 116222652 | Intron-293A>G | G | 0.482 | 0.506 | 0.442 |  | A | 0.475 | 0.815 | 1.000 |
|  |  |  | rs41736 | 116223004 | Coding[59/77]T>C | C | 0.482 | 0.506 | 0.441 |  | T | 0.475 | 0.815 | 1.000 |
|  |  |  | rs41737 | 116223333 | Coding[156/81]A>G | G | 0.482 | 0.506 | 0.442 |  | A | 0.475 | 0.687 | 0.991 |
|  |  |  | rs41738 | 116224442 | 3UTR[1027/1234]A>G | A | 0.482 | 0.506 | 0.442 |  | G | 0.475 | 0.815 | 1.000 |
|  |  |  | rs41739 | 116224740 | 3UTR[1325/936]A>G | A | 0.482 | 0.506 | 0.442 |  | G | 0.475 | 0.902 | 0.993 |
|  |  |  | rs41741 | 116225747 | Flanking_3UTR-71T>G | G | 0.113 | 0.106 | 0.145 |  | G | 0.140 | 0.742 | 0.995 |
|  |  |  | rs6566 | 116225654 | 3UTR[2239/22]A>G | G | 0.482 | 0.506 | 0.442 |  | A | 0.475 | 0.771 | 0.995 |
|  |  |  | rs6951311 | 116221574 | Intron-1371A>G | G | 0.459 | - | 0.500 |  | A | 0.469 | 0.928 | 0.984 |
|  |  |  | rs714180 | 116106238 | Intron-6371A>G | A | 0.470 | 0.471 | 0.442 |  | A | 0.427 | 0.421 | 0.989 |
|  |  |  | rs9641562 | 116110027 | Intron-10160A>C | C | 0.101 | 0.082 | 0.035 |  | C | 0.107 | 0.117 | 1.000 |
| 12 | PTPN11 | protein tyrosine  phosphatase, non-receptor type 11 | rs11066322 | 111406912 | Intron-1750A>G | G | 0.137 | 0.113 | 0.157 |  | G | 0.155 | 0.008 | 0.995 |
|  |  |  | rs12229892 | 111407776 | Intron-886A>G | A | 0.452 | 0.500 | 0.488 |  | A | 0.459 | 0.427 | 0.986 |
|  |  |  | rs2301756 | 111375159 | Intron-223T>C | A | 0.125 | 0.106 | 0.157 |  | T | 0.149 | 0.032 | 1.000 |
|  |  |  | rs4767860 | 111426736 | Intron-146A>G | G | 0.464 | 0.388 | 0.390 |  | G | 0.373 | 0.154 | 0.979 |
|  |  |  | rs7132778 | 111425737 | Intron-1145A>C | C | 0.411 | - | 0.411 |  | C | 0.376 | 0.223 | 0.977 |
|  |  |  | rs7958372 | 111419936 | Intron-4395T>C | C | 0.093 | - | 0.136 |  | C | 0.158 | 0.013 | 1.000 |
| 20 | SRC | v-Src sarcoma  (Schmidt-Ruppin A-2)  viral oncogene homolog | rs12106024 | 35412762 | Intron-4690C>G | G | 0.144 | - | 0.216 |  | G | 0.166 | 0.004 | 0.995 |
|  |  |  | rs12329503 | 35440545 | Intron-3891T>C | C | 0.196 | 0.161 | 0.221 |  | C | 0.163 | 0.232 | 0.947 |
|  |  |  | rs1570209 | 35462245 | Intron-134T>C | C | 0.200 | - | 0.193 |  | C | 0.181 | 0.190 | 0.993 |
|  |  |  | rs16986606 | 35417842 | Intron-9179A>G | G | 0.149 | 0.241 | 0.221 |  | G | 0.170 | 0.001 | 1.000 |
|  |  |  | rs17194717 | 35438267 | Intron-6169C>G | C | 0.167 | - | 0.200 |  | C | 0.143 | 0.003 | 0.848 |
|  |  |  | rs17787897 | 35443621 | Intron-815A>T | A | 0.161 | 0.141 | 0.215 |  | A | 0.147 | 0.007 | 0.850 |
|  |  |  | rs3790150 | 35448513 | Intron-522A>G | C | 0.233 | - | 0.239 |  | G | 0.209 | 0.281 | 0.977 |
|  |  |  | rs6018027 | 35424206 | Intron-2815T>C | C | 0.208 | 0.188 | 0.233 |  | C | 0.157 | 0.387 | 0.984 |
|  |  |  | rs6018088 | 35432039 | Intron-4945A>C | A | 0.167 | 0.135 | 0.209 |  | A | 0.138 | 0.001 | 0.993 |
|  |  |  | rs6018199 | 35443071 | Intron-1365T>G | G | 0.167 | 0.141 | 0.215 |  | G | 0.146 | 0.008 | 1.000 |
|  |  |  | rs6018257 | 35455953 | Intron-38T>C | C | 0.185 | 0.208 | 0.192 |  | C | 0.179 | 0.383 | 0.991 |
|  |  |  | rs6090599 | 35448117 | Intron-126A>G | A | 0.056 | - | 0.034 |  | - | 0.000 | - | 0.000 |
|  |  |  | rs6094509 | 35438689 | Intron-5747A>G | G | 0.173 | 0.141 | 0.209 |  | G | 0.143 | 0.003 | 0.995 |
|  |  |  | rs6122566 | 35468021 | Flanking_3UTR-786A>G | G | 0.179 | 0.265 | 0.174 |  | G | 0.197 | 0.053 | 1.000 |
|  |  |  | rs6124914 | 35449657 | Intron-1666T>C | C | 0.232 | 0.235 | 0.250 |  | C | 0.206 | 0.073 | 0.998 |
|  |  |  | rs6124933 | 35453340 | Intron-2372C>G | C | 0.178 | - | 0.193 |  | C | 0.179 | 0.105 | 0.995 |
|  |  |  | rs744285 | 35443889 | Intron-547T>C | T | 0.151 | - | 0.193 |  | - | 0.000 | - | 0.000 |
|  |  |  | rs747182 | 35416303 | Intron-8231T>C | C | 0.149 | 0.241 | 0.221 |  | C | 0.002 | 0.002 | 1.000 |
|  |  |  | rs8126089 | 35432668 | Intron-5574A>T | T | 0.156 | - | 0.205 |  | T | 0.002 | 0.002 | 0.998 |

* CHB (H): Han Chinese in Beijing, China; CHD (D): Chinese in Metropolitan Denver, Colorado; JPT (J): Japanese in Tokyo, Japan
