# Supplementary figures and images for: Genetic Susceptibility on CagA-Interacting Molecules and Gene-Environment Interaction with Phytoestrogens: A Putative Risk Factor for Gastric Cancer
Source: PLoS One. 2012 Feb 24;7(2):e31020. doi: 10.1371/journal.pone.0031020 (PMC3286459; doi:10.1371/journal.pone.0031020)

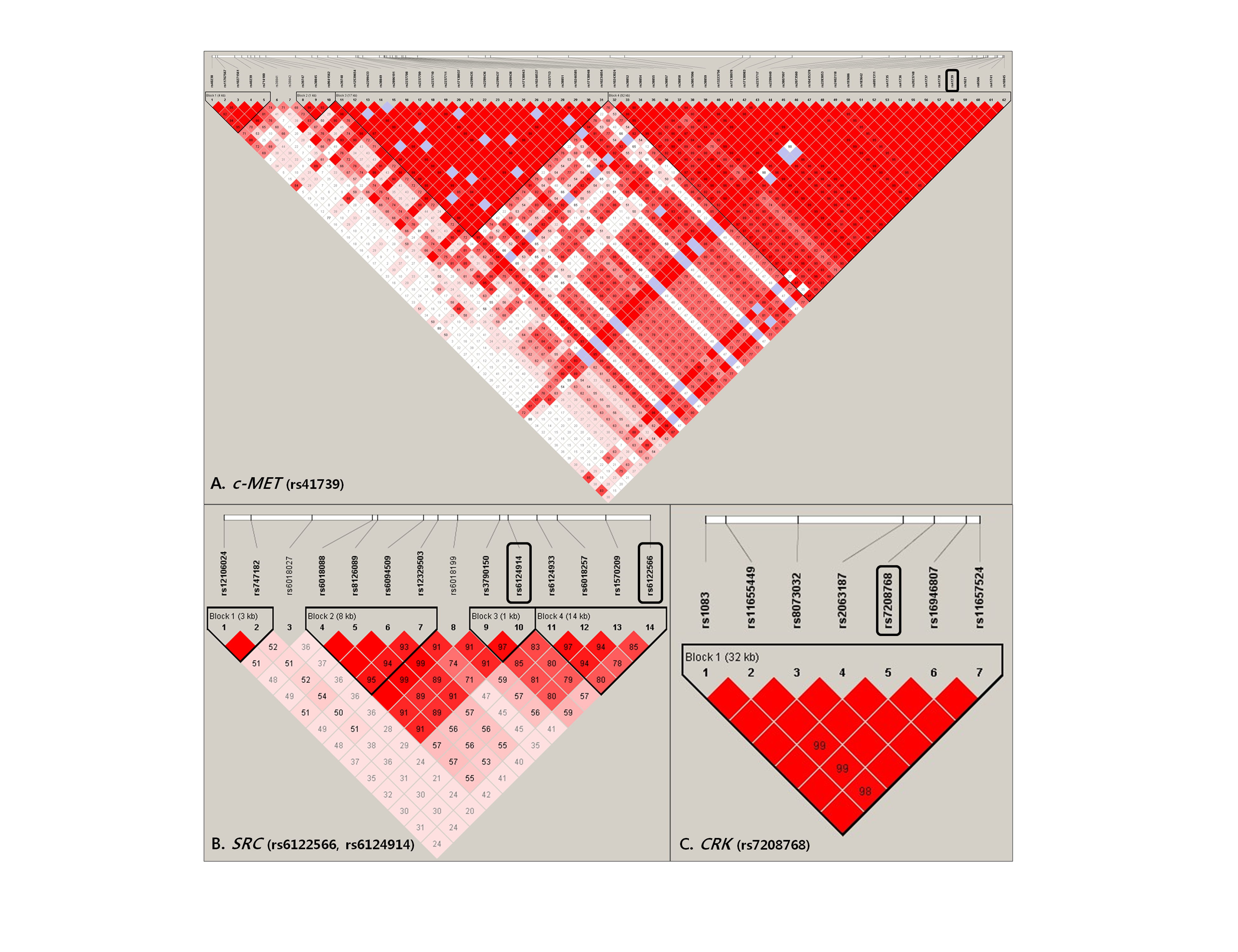

Supplement: Figure S1 — Gene maps and LD blocks. a. D' and LOD values were used for selection of LD color scheme. (TIF) [file pone.0031020.s002.tif]

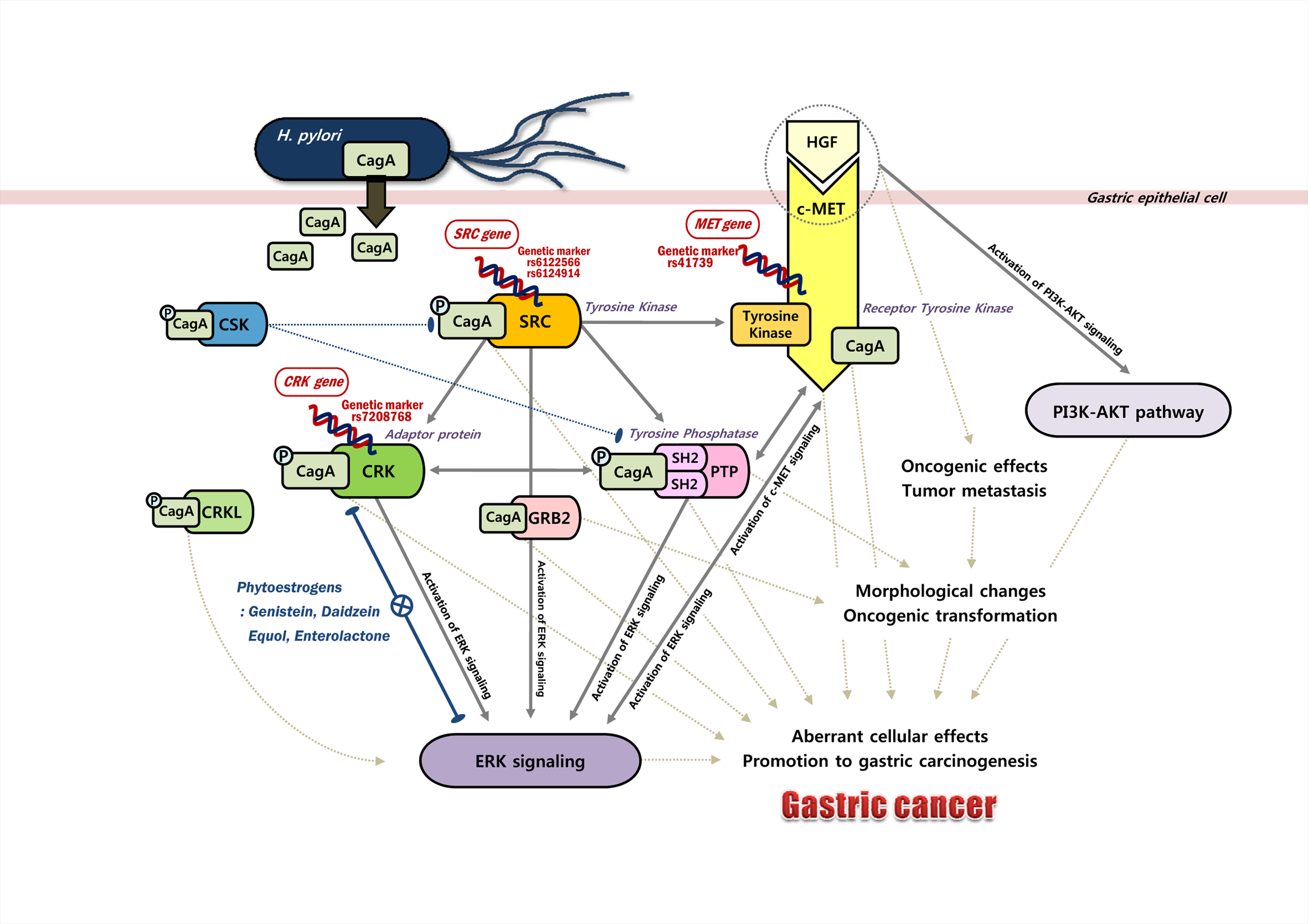

Supplement: Figure S2 — Schematic diagram of oncogenic effects in CagA signal transduction pathway. a. SRC initiates CagA phosphorylation. b. Phosphorylated CagA interacts with CRK adaptor protein and SHP2 (encoded by the PTPN11 gene). c. Non-phosphorylated CagA potentiates c-MET signals and the c-MET-HGF intracellular signaling. d. SRC, c-MET, CRK and SHP2 interacts with phosphorylated or non-phosphorylated CagA to stimulate the ERK cascade linked to aberrant cellular functions that leads to the development of gastric cancer. e. Genetic polymorphisms of SRC (rs6122566 and rs6124914), c-MET (rs41739) and CRK (rs7208768) are significantly associated with gastric cancer risk. f. CSK inhibits SRC family kinase activities and CagA-SHP2 signaling effects. g. Phytoestrogens (Genistein, Daidzein, Equol and Enterolactone) modify the CRK genetic effects. (TIF) [file pone.0031020.s003.tif]
