# Supplementary material for: Genetic Susceptibility on CagA-Interacting Molecules and Gene-Environment Interaction with Phytoestrogens: A Putative Risk Factor for Gastric Cancer
Source: PLoS One. 2012 Feb 24;7(2):e31020. doi: 10.1371/journal.pone.0031020 (PMC3286459; doi:10.1371/journal.pone.0031020)
Supplement: Table S1 — Basic characteristics of gastric cancer cases and controls in the genetic analysis: discovery, extension and pooled analyses. (DOC) [file pone.0031020.s004.doc]

**Table1. Basic characteristics of gastric cancer cases and controls in the genetic analysis: discovery, extension and pooled analyses**

|  |  | **Discovery phase ^a^** | | |  | **Extension phase ^b^** | | |  | **Total gastric cancer cases *vs.* controls** | | |
| --- | --- | --- | --- | --- | --- | --- | --- | --- | --- | --- | --- | --- |
|  |  | **Case**  **(N=76)** | **Control**  **(N=322)** | ***p*** |  | **Case**  **(N=386)** | **Control**  **(N=348)** | ***p*** |  | **Case**  **(N=462)** | **Control**  **(N=670)** | ***p*** |
| **Age** | Mean (SD) | 64.5 (±8.6) | 62.8 (±8.4) | 0.11 |  | 61.5 (±10.5) | 63.1 (±8.4) | 0.02 |  | 62.0 (±10.3) | 63.0 (±8.4) | 0.10 |
| **Sex** | Female | 20 (26.3) | 98 (30.4) | 0.48 |  | 130 (33.7) | 110 (31.6) | 0.55 |  | 150 (32.5) | 208 (31.0) | 0.61 |
| ***H.pylori* infection** | Positive (+) | 64 (84.2) | 271 (84.2) | 0.99 |  | 342 (88.6) | 299 (85.9) | 0.28 |  | 406 (87.9) | 570 (85.0) | 0.18 |
| **CagA** | Positive (+) | 65 (85.5) | 273 (84.8) | 0.87 |  | 355 (92.0) | 308 (88.5) | 0.11 |  | 420 (90.9) | 581 (86.7) | 0.03 |
| **VacA** | Positive (+) | 44 (57.9) | 171 (53.1) | 0.45 |  | 271 (70.2) | 233 (67.0) | 0.34 |  | 315 (68.18) | 404 (60.6) | <0.01 |
| **Smoking status** | Ever smokers **^c^** | 52 (68.4) | 183 (56.8) | 0.06 |  | 238 (61.7) | 191 (54.9) | 0.06 |  | 290 (62.8) | 374 (55.8) | 0.02 |
| **Drink status** | Ever drinkers **^d^** | 46 (60.5) | 184 (57.1) | 0.59 |  | 239 (62.1) | 206 (59.2) | 0.43 |  | 285 (61.8) | 390 (58.2) | 0.22 |
| **Gastric ulcer history** | Positive (+) | 8 (10.5) | 25 (7.8) | 0.38 |  | 59 (17.6) | 50 (18.7) | 0.73 |  | 67 (17.8) | 75 (16.8) | 0.72 |

1. Incidence gastric cancer cases identified in December 2005 and their age, sex, enrollment year and area matched controls from the KMCC
2. Incidence and prevalent gastric cancer cases from the KMCC, incidence gastric cancer cases from Chungnam University Hospital and Hanyang University GURI Hospital and matched cancer-free controls from the KMCC
3. Defined as former and current smokers
4. Defined as former and current drinkers
