# Supplementary material for: Genetic Susceptibility on CagA-Interacting Molecules and Gene-Environment Interaction with Phytoestrogens: A Putative Risk Factor for Gastric Cancer
Source: PLoS One. 2012 Feb 24;7(2):e31020. doi: 10.1371/journal.pone.0031020 (PMC3286459; doi:10.1371/journal.pone.0031020)
Supplement: Table S2 — Significant SNPs for genes which directly encode CagA-binding molecules associated with gastric cancer in the discovery phase. (DOC) [file pone.0031020.s005.doc]

**Table2. Significant SNPs for genes which directly encode CagA-binding molecules associated with gastric cancer in the discovery phase**

| **CHR^a^** | **GENE** | **Selected**  **SNPs ^b^** | **db SNP ID** | **SNP**  **location** | **MAF (%)** | ***Global P* ^c^** | ***P* _permutated_ ^d^** | ***P* _corrected_ ^e,f^** | **OR (95% CI) ^g^** | | |
| --- | --- | --- | --- | --- | --- | --- | --- | --- | --- | --- | --- |
|  |  |  |  |  |  |  |  |  | **Additive ^h^** | **Recessive** | **Dominant** |
| 20 | ***SRC*** | 19 | rs6122566 ^i^ | 3’UTR | G (19.7) | 0.00095 | 0.00284 | 0.0918 | 1.17 (0.76-1.81) | 4.90 (1.19-14.2) | 0.90 (0.53-1.53) |
|  |  |  | rs3790150 | Intron | G (20.9) | 0.04151 | 0.06762 | 0.9610 | 1.57 (1.04-2.37) | 1.04 (0.33-3.21) | 1.98 (1.18-3.32) |
|  |  |  | rs6124914 ^i^ | Intron | C (20.6) | 0.04412 | 0.06904 | 0.9590 | 1.51 (1.01-2.26) | 0.91 (0.30-2.79) | 2.03 (1.21-3.39) |
|  |  |  | rs6018199 | Intron | G (14.6) | 0.04982 | 0.10490 | 0.9813 | 1.52 (0.99-2.35) | 0.63 (0.14-2.88) | 2.00 (1.17-3.40) |
| 7 | ***c-MET*** | 63 | rs41739 ^i^ | 3’UTR | G (47.5) | 0.01275 | 0.00989 | 0.3810 | 1.67 (1.15-2.44) | 1.50 (0.85-2.66) | 2.95 (1.39-6.25) |
|  |  |  | rs16945 | 3’UTR | C (47.5) | 0.01992 | 0.01897 | 0.5805 | 1.62 (1.11-2.36) | 1.50 (0.85-2.65) | 2.65 (1.29-5.46) |
|  |  |  | rs41738 | 3’UTR | G (47.5) | 0.02035 | 0.01686 | 0.5488 | 1.61 (1.11-2.34) | 1.46 (0.83-2.58) | 2.68 (1.31-5.52) |
|  |  |  | rs6566 | 3’UTR | A (47.5) | 0.02045 | 0.01694 | 0.5460 | 1.61 (1.11-2.34) | 1.45 (0.82-2.57) | 2.70 (1.31-5.55) |
|  |  |  | rs10435378 | Intron | G (48.1) | 0.02838 | 0.02392 | 0.6533 | 1.57 (1.08-2.29) | 1.43 (0.81-2.53) | 2.56 (1.24-5.26) |
|  |  |  | rs41737 ^i^ | Exon | A (47.5) | 0.03005 | 0.03528 | 0.7652 | 1.56 (1.08-2.26) | 1.46 (0.83-2.58) | 2.39 (1.19-4.79) |
|  |  |  | rs2023748 | Exon | A (47.5) | 0.03021 | 0.03700 | 0.7567 | 1.56 (1.08-2.26) | 1.46 (0.83-2.58) | 2.39 (1.19-4.80) |
|  |  |  | rs41736 | Exon | T (47.5) | 0.03021 | 0.03700 | 0.7567 | 1.56 (1.08-2.26) | 1.46 (0.83-2.58) | 2.39 (1.19-4.80) |
|  |  |  | rs41735 | Intron | A (47.5) | 0.03021 | 0.03700 | 0.7567 | 1.56 (1.08-2.26) | 1.46 (0.83-2.58) | 2.39 (1.19-4.80) |
|  |  |  | rs6951311 | Intron | A (46.9) | 0.04090 | 0.03994 | 0.8017 | 1.54 (1.06-2.25) | 1.42 (0.79-2.56) | 2.36 (1.17-4.76) |
|  |  |  | rs183642 | Intron | A (47.4) | 0.04090 | 0.03994 | 0.8017 | 1.57 (1.08-2.27) | 1.46 (0.83-2.58) | 2.42 (1.21-4.85) |
|  |  |  | rs2237717 | Intron | T (47.7) | 0.04523 | 0.04891 | 0.8208 | 1.52 (1.04-2.20) | 1.39 (0.78-2.48) | 2.32 (1.16-4.66) |
|  |  |  | rs38859 | Intron | T (48.4) | 0.04821 | 0.06048 | 0.9020 | 1.52 (1.04-2.17) | 2.22 (1.10-4.55) | 1.43 (0.81-2.50) |
| 17 | ***CRK*** | 7 | rs7208768 ^i^ | Intron | A (45.5) | 0.01390 | 0.01392 | 0.3991 | 1.28 (0.90-1.80) | 1.90 (0.89-4.02) | 1.59 (0.87-2.90) |
|  |  |  | rs16946807 | Intron | A (45.6) | 0.02382 | 0.15000 | 0.9972 | 0.98 (0.67-1.42) | 0.51 (0.24-1.09) | 1.52 (0.83-2.80) |
|  |  |  | rs11657524 | Intron | G (45.5) | 0.02679 | 0.15810 | 0.9982 | 0.98 (0.67-1.42) | 0.52 (0.24-1.10) | 1.52 (0.83-2.79) |

1. Chromosome number
2. Total number of SNPs selected within each candidate gene
3. Raw *p*-values calculated by LRT (Likelihood Ratio Test) with 1 degree of freedom in the additive model and 2 degree of freedom in genotypic model
4. Permutated *p*-values calculated from 100,000 permutations in single SNP analysis
5. Corrected *p*-values for multiple comparison with 100,000 permutations in each best model
6. All BH-FDR *p*-values were not significant (*p*>0.05)
7. Adjusted for age, smoking (never *vs.* ever), *H. pylori* infection (positive *vs.* negative) and CagA seropositivity (positive *vs.* negative)
8. Additive effects of allele dosage estimated in the trend model (1 degree of freedom)
9. SNPs selected for the extension analysis
