# Supplementary material for: Genetic Susceptibility on CagA-Interacting Molecules and Gene-Environment Interaction with Phytoestrogens: A Putative Risk Factor for Gastric Cancer
Source: PLoS One. 2012 Feb 24;7(2):e31020. doi: 10.1371/journal.pone.0031020 (PMC3286459; doi:10.1371/journal.pone.0031020)
Supplement: Table S3 — Association between representative SNPs in CagA-binding molecules and gastric cancer risk. (DOC) [file pone.0031020.s006.doc]

**Table 3. Association between representative SNPs in CagA-binding molecules and gastric cancer risk**

|  |  | **Discovery phase** | |  | **Extension phase** | |  | **Total gastric cancer cases *vs.* controls** | | | |
| --- | --- | --- | --- | --- | --- | --- | --- | --- | --- | --- | --- |
|  |  | **MAF ^a^ (%)** | **OR (95% CI) ^b^** |  | **MAF ^a^ (%)** | **OR (95% CI) ^b^** |  | **MAF ^a^ (%)** | **OR (95% CI) ^b,c^** | **OR (95% CI) ^b,d^** | ***P* _heterogeneity_** |
| ***SRC*** | rs6122566 ^e^ | G (19.7) | 4.90 (1.19-14.2) |  | G (17.7) | 4.01 (1.62-9.96) |  | G (18.7) | 3.96 (2.05-7.65) | 4.59 (2.74-7.70) | 0.722 |
|  | rs6124914 | C (20.6) | 1.51 (1.01-2.26) |  | C (18.5) | 1.30 (1.00-1.70) |  | C (19.5) | 1.24 (1.01-1.53) | 1.36 (1.09-1.70) | 0.543 |
| ***c-MET*** | rs41739 | G (47.5) | 1.67 (1.15-2.44) |  | G (48.8) | 1.09 (0.89-1.34) |  | G (48.2) | 1.19 (1.01-1.41) | 1.20 (1.00-1.44) | 0.051 |
|  | rs41737 | A (47.5) | 1.56 (1.08-2.26) |  | A (49.4) | 1.08 (0.88-1.32) |  | A (48.5) | 1.15 (0.97-1.36) | 1.18 (0.99-1.41) | 0.087 |
| ***CRK*** | rs7208768 | A (45.5) | 1.28 (0.90-1.80) |  | A (43.1) | 1.33 (1.08-1.64) |  | A (44.3) | 1.37 (1.15-1.62) | 1.32 (1.10-1.57) | 0.853 |

1. Minor allele frequency among controls
2. All ORs were adjusted for age, smoking (never *vs.* ever), *H. pylori* infection (positive *vs.* negative) and CagA seropositivity (positive *vs.* negative)
3. Pooled analysis including all gastric cases and controls from each study dataset
4. Meta analysis using fixed effect model for combined analysis
5. Estimated in recessive models
