# Supplementary material for: Genetic Susceptibility on CagA-Interacting Molecules and Gene-Environment Interaction with Phytoestrogens: A Putative Risk Factor for Gastric Cancer
Source: PLoS One. 2012 Feb 24;7(2):e31020. doi: 10.1371/journal.pone.0031020 (PMC3286459; doi:10.1371/journal.pone.0031020)
Supplement: Table S4 — The gastric cancer risk associated with the interaction between SNPs in CagA-binding molecules and phytoestrogens. (DOC) [file pone.0031020.s007.doc]

**Table 4. The gastric cancer risk associated with the interaction between SNPs ^a^ in CagA-binding molecules and phytoestrogens ^b^**

|  |  |  |  | **Low level** |  | **High level** |  | ***P***  ***interaction*** |
| --- | --- | --- | --- | --- | --- | --- | --- | --- |
| **Phytoestrogen ^c^** | **Gene** | **db SNP ID** | **Risk allele** | **OR (95% CI) ^d^** |  | **OR (95% CI) ^d^** |  |
| **Genistein** | ***SRC*** | rs6122566 | G | 1.21 (0.86-1.69) |  | 1.06 (0.72-1.54) |  | 0.6461 |
|  |  | rs6124914 | C | 1.43 (1.00-2.03)^**^ |  | 1.31 (0.91-1.90) |  | 0.7173 |
|  | ***c-MET*** | rs41739 | G | 1.21 (0.91-1.60) |  | 1.29 (0.97-1.71)^*^ |  | 0.7958 |
|  |  | rs41737 | A | 1.21 (0.91-1.59) |  | 1.18 (0.90-1.56) |  | 0.9114 |
|  | ***CRK*** | rs7208768 | A | 1.91 (1.44-2.52)^***^ |  | 0.86 (0.63-1.18) |  | 0.0001 |
| **Daidzien** | ***SRC*** | rs6122566 | G | 1.02 (0.66-1.57) |  | 1.24 (0.91-1.71) |  | 0.5401 |
|  |  | rs6124914 | C | 1.33 (0.85-2.09) |  | 1.42 (1.03-1.94)^**^ |  | 0.9133 |
|  | ***c-MET*** | rs41739 | G | 1.24 (0.88-1.74) |  | 1.30 (1.01-1.68)^**^ |  | 0.7950 |
|  |  | rs41737 | A | 1.23 (0.87-1.73) |  | 1.23 (0.96-1.58) |  | 0.9794 |
|  | ***CRK*** | rs7208768 | A | 2.09 (1.46-3.01)^***^ |  | 1.02 (0.79-1.32) |  | 0.0013 |
| **Equol** | ***SRC*** | rs6122566 | G | 1.32 (0.79-2.22) |  | 1.28 (0.93-1.77) |  | 0.7984 |
|  |  | rs6124914 | C | 2.78 (1.50-5.16)^**^ |  | 1.27 (0.93-1.75) |  | 0.0165 |
|  | ***c-MET*** | rs41739 | G | 1.07 (0.75-1.53) |  | 1.50 (1.14-1.96)^**^ |  | 0.1563 |
|  |  | rs41737 | A | 1.07 (0.75-1.53) |  | 1.40 (1.07-1.82)^**^ |  | 0.2641 |
|  | ***CRK*** | rs7208768 | A | 1.87 (1.26-2.78)^**^ |  | 1.06 (0.82-1.39) |  | 0.0147 |
| **Enterolactone** | ***SRC*** | rs6122566 | G | 0.88 (0.50-1.57) |  | 1.31 (0.98-1.75)^*^ |  | 0.3222 |
|  |  | rs6124914 | C | 1.64 (0.85-3.14) |  | 1.38 (1.04-1.84)^**^ |  | 0.4381 |
|  | ***c-MET*** | rs41739 | G | 0.93 (0.59-1.47) |  | 1.34 (1.06-1.70)^**^ |  | 0.1899 |
|  |  | rs41737 | A | 0.92 (0.59-1.44) |  | 1.29 (1.02-1.62) |  | 0.2356 |
|  | ***CRK*** | rs7208768 | A | 1.77 (1.10-2.85)^**^ |  | 1.07 (0.84-1.35) |  | 0.0404 |

*** *p* <0.0001, ** *p* <0.05, * 0.05≤ *p* <0.10

1. Additive effects of allele dosage estimated in the additive model
2. Cut-off level of each biomarker was decided using Spline analysis
3. Mean of biomarkers among low and high concentration group: genistein 73.7 nmol/L *vs.* 312 nmol/L; daidzein 13.8 nmol/L *vs.* 168.3 nmol/L; equol 3.4 nmol/L *vs.* 86.5 nmol/L; enterolactone 4.0 nmol/L *vs.* 85.5 nmol/L
4. Adjusted for age, smoking (never *vs.* ever), *H. pylori* infection (positive *vs.* negative) and CagA seropositivity (positive *vs.* negative)
